# Decoding ladybird’s colours: Structural mechanisms of colour production and pigment modulation

**DOI:** 10.1371/journal.pone.0324641

**Published:** 2025-06-11

**Authors:** Marzia Carrada, Mohamed Haddad, Luis M. San-Jose, Gonzague Agez, Jean-Marie Poumirol, Alexandra Magro

**Affiliations:** 1 Centre de Recherche sur la Biodiversité et l’Environnement (CRBE), Université de Toulouse, CNRS, IRD, Toulouse INP, Toulouse, France; 2 CEMES-CNRS, Université de Toulouse, Toulouse, France; 3 PHARMA-DEV, Faculté de Pharmacie, Université de Toulouse, Toulouse, France; 4 Université de Toulouse – ENSFEA, Castanet-Tolosan, France; Central Food Technological Research Institute CSIR, INDIA

## Abstract

This study investigates the mechanisms underlying colour production in the family Coccinellidae, focusing on two model species: *Adalia bipunctata* (L.) and *Calvia quatuordecimguttata* (L.). In this family, colours have traditionally been attributed primarily to pigments such as carotenoids and melanins. We propose an alternative perspective, considering the elytra as an integrated optical medium whose optical properties – and hence colouration – result from both its architectural design and the properties of its constituent materials, including matrix and pigments. In the present work, the elytron microstructure was precisely determined by transmission electron microscopy and the numerical replica was then injected into numerical simulations of the microstructure’s interaction with light, showing that the elytron structure is able to select a range of wavelengths and then generate colour. Coupling these results with local pigment analyses and microstructural examination of elytra, we show that while pigments are central to patterning and contribute to colour, the overall colour also results from one or more physical mechanisms that may operate simultaneously. In the light of these results, we suggest that the complex and diverse colouration in the Coccinellidae can only be elucidated by considering the interplay of pigments and the optical properties of the elytron cuticle. From an evolutionary ecology point of view, elytra structure influence on colouration may provide new insights into colour signalling in this insect family.

## Introduction

Colouration displayed by animals has been the target of intense research aiming at understanding how it is produced, both proximately (*i.e.*, its developmental, genetic and morphological determinants) and ultimately (*i.e.*, its adaptive role and its evolutionary origin and stability) [1]. The pigmentary origin of colouration has received a predominant attention probably because pigments’ inherent properties (namely pleiotropic effects on individuals’ physiology and behaviour) have allowed biologists to link colourful displays to their signalling role in distinct contexts such as reproduction or aposematism [2–4]. However, pigments are likely to explain only a small fraction of nature’s colour palette [5,6]. Moreover, pigments must rely on underlying, often complexly elaborated, reflective structures to produce any colouration [7,8]. Although the structural part of colouration is gaining recognition [9–11], its contribution to the production and the signalling function of colour traits is largely neglected, particularly when pigments are also present in the coloured integument. Structural colouration has received less attention comparatively to pigment-based colouration for different reasons. First, structural components, contrary to pigments, lack a clear framework explaining how they can signal genetic or phenotypic quality. In the same line, the genetic pathways building up structural colourations are poorly known compared to those of pigments [12] even in well-studied taxa such as birds [13]. Nevertheless, evidence showing that structural colouration does associate with quality aspects such as individual condition accumulates in different taxa [14,15]. Secondly, the study of structural colouration requires a good understanding of the physics of light and how small changes in integumentary structures translate into colour changes. Several physical mechanisms may occur at the same time and the light interaction with the structure becomes very complicated and hard to describe [16]. As a consequence, structural and pigment-based colourations have often been studied in isolation [17], maintaining a long-standing dichotomy that classifies colour traits as either strictly structural or pigment based [18]. This established dichotomy contrasts with the reality that both pigments and structures contribute to reflectance spectra and both influence the overall optical properties of the integument, i.e., properties defining its interaction with light. Indeed, it is important to bear in mind that colouration is a perceptual phenomenon rather than a physical quantity, but it can be quantified through a measurement of the reflectance spectrum of the elytra. The CIE 1931 chromaticity coordinates, representing a standard human observer’s perception, can be derived from the reflectance spectra by multiplying with Planck’s spectral luminance function at 6000K and applying the CIE 1931 colour matching functions.

Insects have been central to the study of animal colouration. However, structural and pigment-based colourations in insects have been most often considered separately. In the highly diversified Coleoptera order, for instance, structural colouration has been intensively studied in certain families, such as in the Scarabaeidae, that display marked iridescent colours, or the Chrysomelidae. Vigneron et al. [19] investigated the vivid and iridescent blue colouration of *Hoplia coerulea* (Drury) (Coleoptera: Scarabaeidae) and attributed it to the layer-by-layer structure of the beetle cuticular squamule, that act like natural photonic devices. Vigneron et al. [20] also studied the tortoise beetle *Charidotella egregia* (Boheman) (Coleoptera: Chrysomelidae), which is able to modify its structural colour under stress conditions thanks to humidity content variation in a chirped (*i.e.,* showing a progressive decrease of the layers thickness) multilayer reflector. Another chirped multilayer reflector producing gold colouration was observed by Pasteels et al. [21] in another species of the same genus, *C. ambita* (Champion). Although these examples testify of structural based colouration research in Coleoptera, such studies have been neglected in some Coleopteran families, where pigments have received most attention. This is the case of the Coccinellidae family, commonly known as ladybirds or ladybugs, a highly diversified family with 6000 described species [22] which harbour a wide range of colours and patterns but whose colouration has been exclusively considered from the perspective of its pigmentary origin.

Melanins and carotenoids are pigments considered to underlie the iconic red with black spots colouration of many species of ladybirds [23]. The variation in ladybird colouration, ranging from beige to black and encompassing all shades of red-orange, may partly explain why pigments have received greater attention in the literature thus far. Furthermore, it is noteworthy that the most studied structural colours are green, blue or metallic (e.g., silver or gold), while red hues are more commonly attributed to pigments, even if structural iridescent red has been documented [24]. Ladybirds’ elytra are hardened fore wings and have the typical laminar structure of insect cuticle, which is essentially composed of structural proteins and chitin but also contains water and in some cases pigments and metals [25–28]. Their mechanical properties have been investigated by Zhou et al. for *Coccinella septempunctata L.* [29], but studies on ladybirds’ elytra are not extensive and, to our knowledge, there is no research work on the structural colouration of ladybirds.

Taking all the above into consideration, we defend that investigating the origin of colour and the evolution of colour-related interactions in ladybirds requires a broader perspective that includes not only pigments but structure as well. To test this hypothesis, in this work we investigate the origin of colour in two species, *Adalia bipunctata* (L.) and *Calvia quatuordecimguttata* (L.), which are different in terms of elytra colour and patterns.

*A. bipunctata* is polymorphic regarding to elytra pattern, and presents forms ranging from red to almost completely black elytra [30] (in the present study we analysed individuals from the “typica” morph. *C. quatordecimguttata,* on the other side, is monomorphic and the elytra appear brown with cream spots, the colouration of which cannot be solely attributed to pigmentation as no known pigment is capable of producing the glossy white/ creamy hue observed. Despite their phenotypic differences, the two species belong to the same tribe, the Coccinellini [22], are of Paleartic origin [31], are present in the same habitats, and commonly co-occur in communities of aphidophagous Coccinellidae [32]. That is, they thrive in similar light and temperature contexts. Moreover, in the laboratory they can be reared exclusively on the same aphid species *Acyrthosiphon pisum* (Harris) [33,34] enabling control over their carotenoid’s food source.

Understanding how structure and pigments interact with light and contribute to the observed colouration in ladybirds, requires investigating both their physical and chemical properties. In the present work, several methods were used, ranging from optical and electron microscopies, light-based techniques, to LC-HRMS analyses.

## Materials and methods

### Ladybird culture

**Fig 1:**
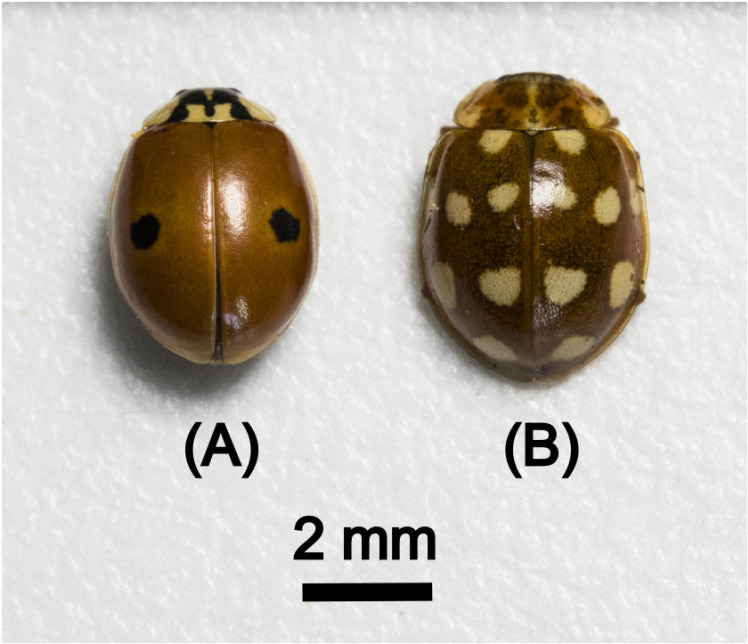
Photographs of an individual of both the studied species. Colouration and patterns of adults of (A) *Adalia bipunctata* (L), “typica” morph and (B) *Calvia quatuordecimguttata* (L).

Fig 1 shows photographs of an individual of both the studied species, Adalia bipunctata (L.) (A), and Calvia quatuordecimguttata (L.) (B). Individuals of the two species were obtained from stock cultures maintained at the “Centre de Recherche sur la Biodiversité et l’Environnement” Laboratory (Toulouse, France). These consisted of adults reared at 21 ± 1°C, LD 16:8, in 5-litre plastic boxes with a piece of corrugated filter paper, on which the females laid eggs. Three times a week they were fed an excess of pea aphids, Acyrthosiphon pisum Harris. Two stems of broad bean, Vicia faba L., were added to each box to improve the survival of the aphids. Eggs were taken from the stock cultures every rearing day and incubated in 175 cm3 plastic boxes kept under the same conditions as described above. After hatching, larvae were fed excess pea aphids three times a week and reared to adult stage.

Adults studied in this work were between 15 and 20 days old and have developed their characteristic colouration. They were collected from the stock cultures, frozen and kept at – 21°C until analyses, in order to avoid the degradation at room temperature and air ambient. It should be noticed that preliminary results have shown that the elytra are stable for at least three weeks at room temperature and no difference was found between the results of stored and immediately processed samples. Just before the analyses, the elytra were removed with the help of a scalpel.

### Physical properties

#### Optical and electron microscopies.

The elytra were analysed both at the surface and cross section levels. The elytra surface was first observed at the micron meter scale by a Nikon Eclipse LV100ND optical microscope in reflection mode and then analysed using Scanning Electron Microscopy (SEM), at nanometric scale, with a Helios NanoLab600i SEM. Other than allowing to image the surface at bigger magnification than optical microscopy, SEM detects morphology independently from light interaction with the samples.

In parallel, Transmission Electron Microscopy (TEM) was used in cross sections of elytra in order to investigate the elytra microstructure up to nanometric scale, and to identify the phenomena that could be responsible for their physical colour. Cross-sectional samples for TEM were prepared in CMEAB (Centre de Microscopie Electronique Appliquée à la Biologie, Toulouse, France). The sample preparation protocol differed between the two species. In the case of *C. quatuordecimguttata,* the elytra were fixed in 2% glutaraldehyde in 0.1 M Sorensen phosphate buffer (pH 7.4) for 4 hours at 4°C, followed by a 12-hour wash in 0.2 M phosphate buffer at 4°C. The samples were then post-fixed for 1 hour at room temperature in 1% osmium tetroxide with 250 mM sucrose and 0.05 M phosphate buffer. Dehydration was carried out through a graded ethanol series, followed by propylene oxide treatment, before embedding in Embed 812 resin (Electron Microscopy Sciences). In contrast, *A. bipunctata* elytra were directly embedded in the same resin, bypassing the preparatory steps required for *C. quatuordecimguttata* elytra, where direct resin embedding caused elytra tissue to tear and fold in during cutting, preventing observation. Polymerisation of the resin blocks was achieved over 48 hours at 60°C.

Finally, the elytra were sectioned into 70 nm-thick slices using an Ultracut Reichert Jung ultramicrotome fitted with a diamond knife and mounted on 100-mesh collodion-coated copper grids.

SEM and TEM images were analysed using the software ImageJ [35], version 2.14.0/1.54f.

#### Light based methods.

Local measurements for reflectance spectra were performed in order to quantify the elytron colouration and investigate the light interaction with the elytra surface. Raman spectroscopy was used as a non-destructive method to detect the presence of pigments in the elytra. This is a physical method to detect chemical molecules vibration energies based on the inelastic scattering of light by molecules and has been already employed to characterize pigments (carotenoids, melanin…) present in different animal tissues [36–38]. As Raman peaks provide direct information about the vibrational energies of molecules, pigments can be identified by comparison with specific spectra reported in the literature or with commercial standard pigments. For these measurements, a X-Plora Horiba/Jobin Yvon device was used with a confocal microscope, a white light lamp, a 532 nm laser wavelength, and a 100X objective lens. Several gratings are available with 2400, 1800, 600 and 300 gr/mm (grooves per mm). Increasing the number of grooves/mm increases spectral resolution but reduces the observed spectral range and the signal intensity, so the right configuration has to be chosen on a case-by-case basis. In the present study, the 300 gr/mm grating was chosen for white light experiments (*i.e.,* reflectance spectra recording), in order to obtain the larger spectral range and the 1800 gr/mm was used for Raman spectroscopy. To acquire reflectance spectra for the elytra, the reflected spectra registered in the direction of the microscope axis (normal reflection) was divided by a reference spectrum, corresponding to a perfect mirror in the visible range (100% reflector).

For all the analyses, 10 elytra from 10 different individuals of each species were studied. All the spectra were recorded in the same area of the elytra, close to the black or creamy spots depending on the species, and with the laser beam perpendicular to the elytron surface.

#### Numerical simulations.

Numerical simulations were conducted using the finite-difference time-domain (FDTD) method, implemented with the Meep software package [39]. This electromagnetic simulation tool solves Maxwell’s equations iteratively over time within a finite computational region. The FDTD approach was chosen due to its accuracy in predicting the optical response in inhomogeneous multilayer structures [40]. In particular, the thicknesses of each interlayer were adjusted to replicate the contrast alternation observed in the TEM cross-sectional images.

The computational grid resolution was set to 100 pixels per μm, with an additional subpixel smoothing routine. Absorbing boundary conditions were employed at the edges of the computational domain to minimize reflection artefacts. These conditions were achieved using a perfectly matched layer (PML) setup, which surrounds the computational cell with a non-reflective medium that absorbs outgoing electromagnetic waves.

To explore the optical properties of the system, we set the optical birefringence between layers to 0.15, while scanning the mean refractive index between 1.30 and 1.55, without any absorption (k = 0). The incident light was generated from point sources randomly distributed in front of the structure, simulating a wide range of incidence angles. The broadband, incoherent electromagnetic wave source was designed to emulate natural daylight conditions. Reflectance spectra were obtained as the average of ten independent computational runs, each with slight random variations (with a standard deviation of 5 nm) in the geometry to account for inherent variability in the biological structure.

For chromaticity response analysis, each reflectance spectrum was multiplied by Planck’s spectral energy luminance function at 6000K in order to obtain the spectral radiance of the system under white light illumination. Then, we employed the CIE 1931 colour matching functions, which use a set of tristimulus values that correspond to the spectral sensitivities of the three types of cone cells in the human eye. This approach allowed for an accurate assessment of the perceived colour response of the simulated structures under daylight conditions (see S1 File for colour reproduction of a photographic standard colour chart).

### Chemical analyses

#### Sample preparation and extraction.

Ladybird elytra samples (6 replicates, 2 mg each) were extracted using a solvent mixture of ethanol:hexane:MTBE (1:0.5:0.5). Samples were homogenized using a FastPrep-24 instrument (3 cycles of 20s at 4 ms − 1, with 5-min cooling periods), then centrifuged (15 min, 3500 rpm, 4°C). The supernatants were collected and stored at 4°C. A quality control sample was created by pooling aliquots from all extracts for LC-HRMS analysis.

#### UHPLC–HRMS profiling, peak analysis, features identification.

See S2 File.

### Statistical analysis

Statistical analysis was done by uploading the.csv file to the Metaboanalyst platform version 5.0 (https://www.metaboanalyst.ca/). The data were pre-processed, first by normalization (by median) of the samples, then the variables were weighted by auto scale (mean-centered and divided by the square root of the standard deviation of each variable). A Principal Component Analysis (PCA) then a Partial Least Squares Discriminant Analysis (PLS-DA) were carried out. Finally, the m/z and the retention time of the significant features associated with their normalized peak area were plotted on a heat map (hierarchical clustering) using ANOVA. To test the significance of differences between two-group data (Adalia vs Calvia) t-tests (p-value < 0.05) were performed.

## Results

**Fig 2:**
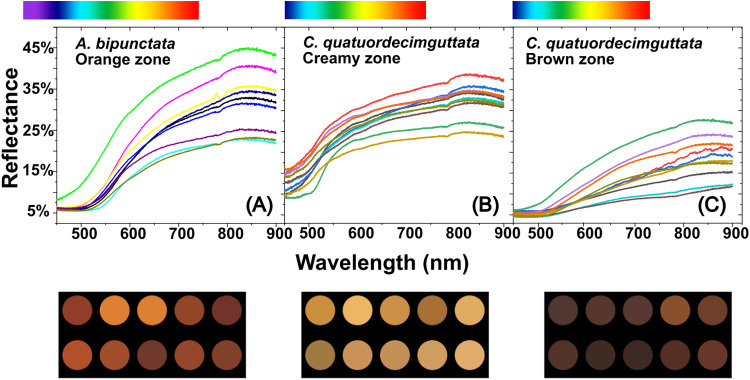
Reflectance spectra. The curves are the reflectance spectra of ten analysed individuals for each species and obtained for (A) the orange zone of *A. bipunctata* elytra and the creamy (B) and brown (C) zones of *C. quatuordecimguttata* elytra. A (human) colour scale corresponding to wavelengths axis is placed over each spectrum. The colour patches at the bottom of each figure correspond to the colours that are perceived for each of the reflectance spectra.

*A. bipunctata* elytra appear orange-reddish with black spots, while those of *C. quatuordecimguttata* appear brown with creamy spots. The presence of iridescence, defined as changes in hue that are perceived by the human eye depending on the angle of observation or illumination, is not evident in either species under investigation. Fig 2 illustrates the reflectance spectra and the associated colour patches obtained for (A) the orange zone of *A. bipunctata* elytra and the creamy (B) and brown (C) zones of *C. quatuordecimguttata* elytra, respectively.

Reflectance spectra for the black spots of *A. bipunctata* are not shown due to the extremely low signal detected. For all the other observed elytra zones, light is predominantly reflected within the yellow-to-red range although maximum reflectance occurs in the infrared, which is invisible to human beings, insects, and birds [41] (a colour scale corresponding to wavelengths perceived by humans is added to each figure for clarity). Thus, the lowest reflectance values for the three observed zones are between 400 and 500 nm, which correspond to the spectral zone where carotenoids typically absorb light [33]. The perceived colour can be determined as described in the section “Numerical Simulations” by multiplying each reflectance spectrum by Planck’s spectral energy luminance function at 6000K and using the CIE 1931 colour matching functions. This results in the coloured patches at the bottom of the Fig 2A–2C, which correspond to the ten reflectance spectra recorded from each elytra zone. Variations between individuals are observed in both chromaticity and luminance, with luminance referring to the light intensity, which is associated with the amount of reflected light. It is important to note that the measurements were taken at a highly localised scale (using a x100 lens), although the numerical aperture of the lens allows for light collection across a broad angle. Consequently, the coloured patches do not represent a uniform colouration across the entire elytron, and variations may occur even within a single elytron.

**Fig 3:**
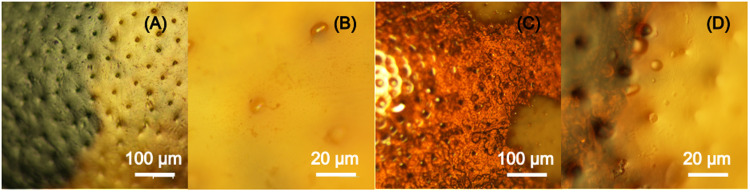
Optical microscopy. Optical microscope images of a same zone of the elytron surface of an *A. bipunctata* specimen (A and B) and of a *C. quatuordecimguttata* specimen (C and D).

When the elytra of the two species are observed with the optical microscope in reflection mode, small-scale differences are detected in their morphology and colouration (Fig 3).

*A. bipunctata* elytra present a rather regular surface colour, bearing concavities evenly distributed in the orange and black zones (Fig 3A and 3B). Contrarily, the colour of *C. quatuordecimguttata* elytra’s surface is not regular (Fig 3C). Whereas it is uniformly coloured in the bright/creamy spot zones, the brown zones are formed by red-brown and darker grains. As for *A. bipunctata*, concavities are also present and evenly distributed in the brown and creamy zones (Fig 3D). These cavities have a diameter of more than 5 ηm and cannot be involved in colour production, as evidenced by the fact that, in both species, the zones between them are orange, black, brown, or creamy depending on the elytron zone.

**Fig 4:**
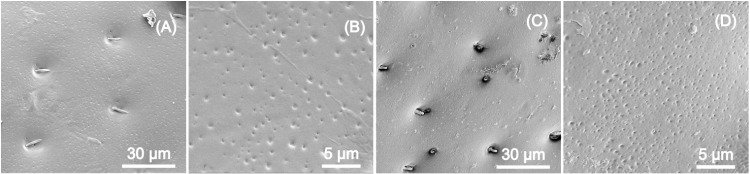
Scanning electron microscopy. SEM images of the surface of an *A. bipunctata* (A and B) and *of a C. quatuordecimguttata* elytra (C and D).

The Scanning Electron Microscopy (SEM) images of the two species’ elytra reveal that concavities are in both cases seta-bearing (Fig 4A and 4C) and also show the existence of smaller concavities in between the larger ones (Fig 4B and 4D).

These smaller cavities have a diameter between 125 and 370 nm, and 75 and 400 nm respectively for *A. bipunctata* and *C. quatuordecimguttata.* Using the Euclidean Distance Measurements (EDM) approach in the Fiji (ImageJ) program, the mean distance between these smaller concavities is 814 nm ± 440 nm for the first species whereas it is 173 nm ± 100 nm for the second one.

**Fig 5:**
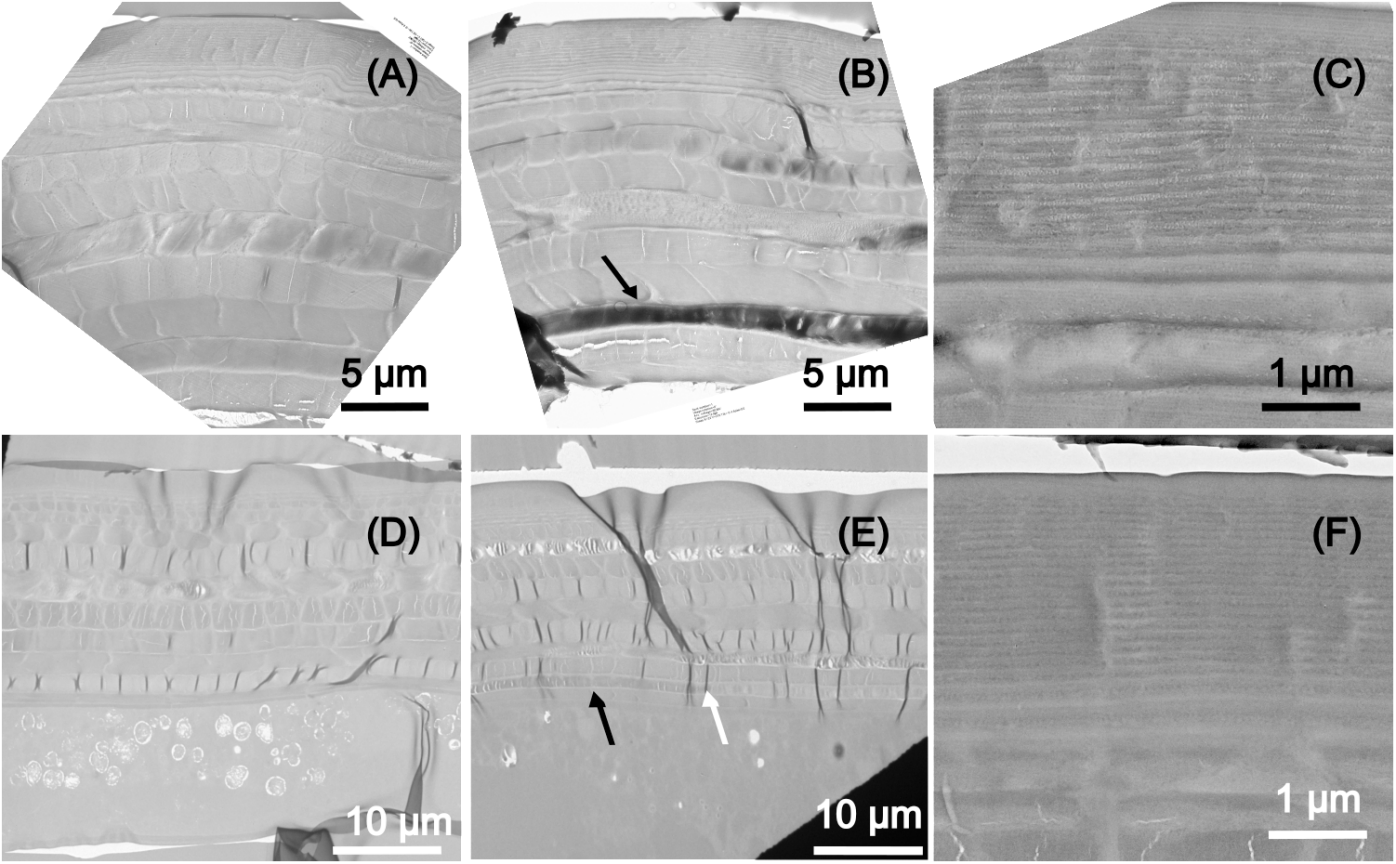
Transmission electron microscopy. TEM images of the cross-section microstructure of (top raw) an elytron of *A. bipunctata* (A) section of an orange zone, (B) section of a black spot zone, (C) zoom on the exocuticle multilayer, and of (bottom raw) an elytron of *C. quatuordecimguttata* (D) brown zone, (E) transition from brown and creamy spot zone, (F) exocuticle multilayer. Black arrows in panels B and E indicate the location of an electron high absorbing layer. The white arrow in panel E indicates the location of the frontier between the zones with and without the electron high absorbing molecule.

Fig 5 depicts the Transmission electron microscopy (TEM) cross-section microstructure of the elytra of the two species.

In both species the endocuticle presents a laminar structure made up of multilayers of darker and brighter contrast (Fig 5A and 5D). In the case of *A. bipunctata* these layers are 1–2 ηm thick whereas in *C. quatuordecimguttata* they range from 1 to 2.2 ηm thick. It should be noted that the cross-section microstructure of a black spot zone of the *A. bipunctata* elytra (Fig 5B) shows an electron-high absorbing layer (black arrow), indicating the presence of a molecule that is opaque to electrons, potentially eumelanin. A similar yet less contrasted layer is observed in the brown but not creamy zones of the *C. quatuordecimguttata* elytra (see black arrow in Fig 5E). Finally, the epidermis of *C. quatuordecimguttata* elytra is larger than that of the *A. bipunctata* elytra. The exocuticle of *A. bipunctata* examined at higher magnification corresponds to a band made of 30 thin layers, with a period spanning from 112 to 270 nanometres, and it has the same properties (total thickness and periods) on the orange and black zones. Furthermore, this exocuticle multilayer reveals the presence of several nanoparticles (30–40 nm diameter) within the thin layers (see Fig 5C). The exocuticle has been found to have the same structure for all the observed specimens. The exocuticle of *C. quatuordecimguttata* is also organised in a chirped multilayered structure, but it is made of 42 layers with periods varying from 103 nm to 270 nm. Nanoparticles are also visible in this layer, with 25–40 nm diameter (Fig 5F) and porosities of the same order of magnitude can be observed. In this species the exocuticle also has the same properties (total thickness and periods) on the brown and creamy zones, as shown in the Fig 5E, depicting the frontier (white arrow) between the brown zone of the elytron and a creamy spot.

**Fig 6:**
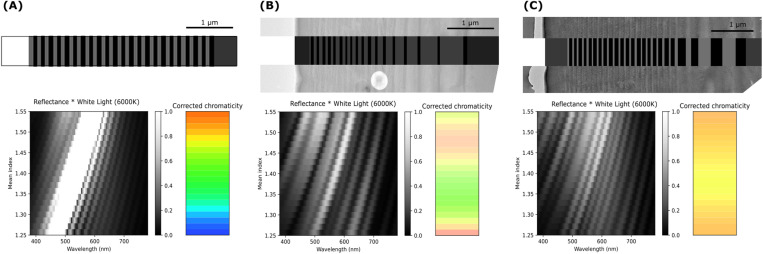
Simulated structural chromaticity. Top row: (A) Bragg grating with a fixed periodicity p = 190 nmp = 190 nm; (B) Correspondence between the TEM cross-sectional image of the *A. bipunctata* elytra structure and its numerical replica; (C) Similar analysis for *C. quatuordecimguttata*. The grayscale represents variations in the refractive index. Bottom row: Dependence of reflection radiance on the mean refractive index and the corresponding CIE 1931 chromaticity coordinates.

The interaction of light with the chirped multilayer in the exocuticle of ladybirds has been modelled in accordance with the methodology outlined in the section “Numerical Simulations”. Fig 6 presents the simulated structural chromaticity, excluding the effects of light absorption from pigments and/or the matrix.

Initially, a model Bragg grating with a fixed periodicity approximating that observed in the ladybird multilayers (p = 190 nm, n = 190 nm) was evaluated. This analysis demonstrated that such a structure is capable of selectively reflecting colour across the visible light spectrum, contingent on the mean refraction index. Subsequently, the numerical replica of the structures corresponding to the TEM cross-sectional images of the *A. bipunctata* (B) and *C. quatuordecimguttata* (C) elytra were analysed. The dependence of reflection radiance on the mean refractive index was calculated for each species, and the corresponding CIE 1931 chromaticity coordinates were determined. These simulations suggest that the structural characteristics of *A. bipunctata* would give rise to a colouration ranging from green to orange-pink, whereas those of *C. quatuordecimguttata* would produce a colouration that ranges from yellow to orange. The observed colouration is primarily determined by the average refractive index of the chitin matrix. However, the presence of pigments may lead to the absorption of certain reflected wavelengths, thereby influencing the final perceived colour. We therefore examined the pigments present in the elytra.

**Fig 7:**
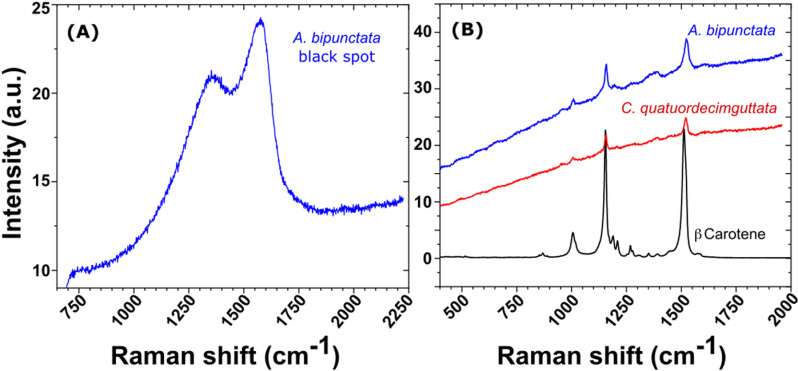
Raman spectra in different elytral zones. Raman spectrum of the black dot of an *A. bipunctata* elytron. (B) superposition of beta carotene reference spectra with the Raman spectra obtained in the orange zone of an *A. bipunctata* and in the brown zone of a *C. quatuordecimguttata* elytra.

Fig 7 shows an analysis of elytra pigment content using Raman spectroscopy for the two species.

**Fig 8:**
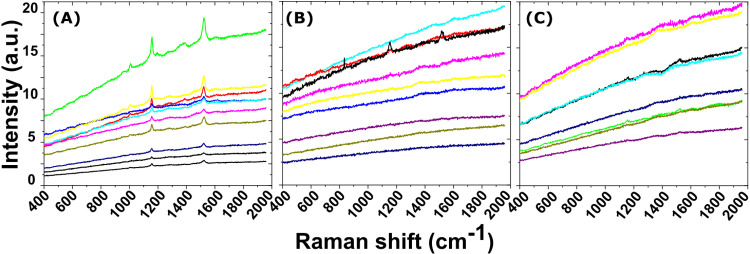
Variability in Raman Spectra. Raman spectra of the elytra of ten specimens of (A) *A. bipunctata* orange zone, and of ten specimens *C. quatuordecimguttata* (B) creamy and (C) brown zones.

Fig 7A depicts a typical Raman spectrum obtained from the black dots on the elytra of *A. bipunctata* ladybirds. The spectrum structure and peak positions correlate to the reported Raman peaks for eumelanin, which are located at 500 cm^-1^, 1380 cm^-1^, and 1580 cm^-1^ [42]. Because the Raman peaks’ positions are determined by the molecule’s surroundings, these values may vary slightly depending on the matrix. In our case the peaks are centred at 1380 cm^-1^ and 1567 cm^-1^. Raman peaks corresponding to eumelanin have not been identified in any additional regions of the elytra, including neither the orange area of *A. bipunctata* nor the brown areas of *C. quatuordecimguttata*. In Fig 7B, the Raman spectra obtained in the orange zone of an *A. bipunctata* elytron and in the brown zone of a *C. quatuordecimguttata* elytron are superposed with beta carotene reference spectra. The highest peaks in all of the spectra correspond to carotenoids. Indeed, the largest contributions of carotenoids to Raman spectra are in the ranges [1000; 1008] cm^-1^ (C-CH3 bonds), [1151; 1156] cm^-1^ (C-C bonds), and [1511; 1529] cm^-1^ (C = C bonds), suggesting the existence of these pigments in the elytra of both species. A study of Raman spectra from ten different individuals of each species confirms that carotenoids are present in both (Fig 8).

However, there is also intraspecific variation within each species. In the case of *A. bipunctata*, Raman spectra (Fig 8A) show very strong carotenoids’ signal, present in all individuals. In *C. quatuordecimguttata,* in contrast, carotenoids signal is very weak and detectable only in 3 out of the 10 analysed individuals, implying that carotenoid concentration can be very low or even absent in some individuals of this species. Interestingly, in the case of *C. quatuordecimguttata*, when carotenoids are present, the corresponding peaks appear in both the brown and creamy zones of the elytron.

**Fig 9:**
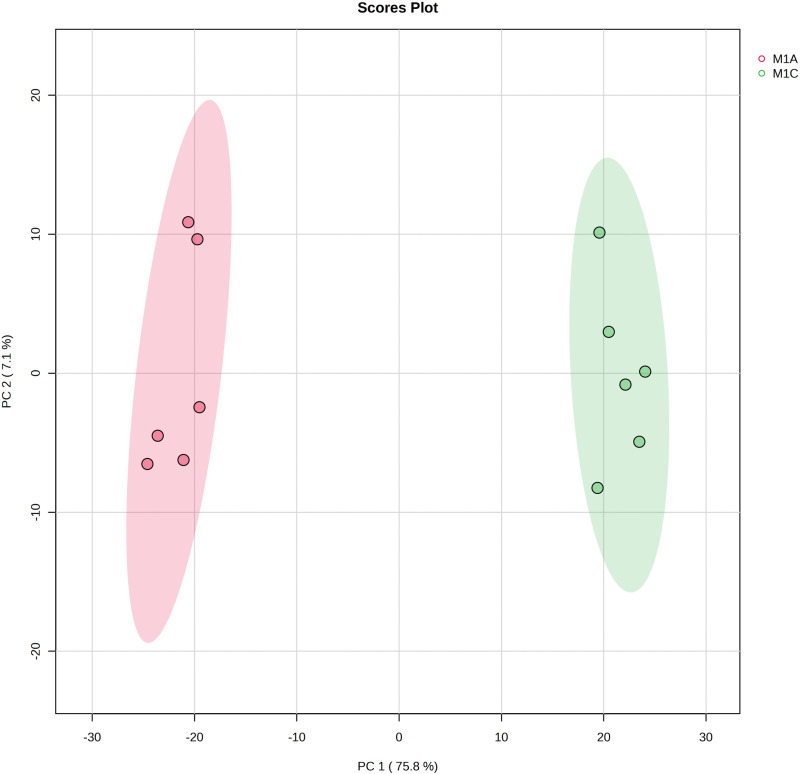
LC-MS Chromatograms. PCA corresponding to the extracts of *A. bipunctata* and *C. quatuordecimguttata* based on the variables (normalized peak area as a function of *m/z* at an Rt) detected on the LC-MS chromatograms.

The metabolomic analysis revealed two clearly distinct clusters corresponding to *A. bipunctata* and *C. quatuordecimguttata* samples (Fig 9), demonstrating a strong differentiation in their overall metabolic profiles.

When focusing specifically on pigment content, chemical analyses of the elytra revealed substantial differences in beta-carotene levels between the two species, confirming the Raman results. A prominent peak corresponding to beta-carotene (m/z 536.43750/Rt 11.413 minutes) was detected in *A. bipunctata*, whereas *C. quatuordecimguttata* samples showed minimal beta-carotene presence.

## Discussion

The traditional approach to the study of animal colouration typically separates pigmentary and structural origins. However, we argue that this separation is potentially misleading. Pigments do not exist in isolation; they are invariably embedded within a matrix – the primary constituent of the tegument – which possesses intrinsic optical properties that both modulate the absorption characteristics of the pigments and are influenced by them in return. Structural colouration arises from the design of the tegument (such as its periodicity, size, and organisation) but it also depends on the material’s intrinsic properties, particularly its ability to refract light, quantified by the refractive index n which is a characteristic of the material and varies with wavelength. For absorbing materials, the refractive index is a complex number N = n + ik, where the imaginary component k accounts for light attenuation due to absorption. Both pigments and the matrix material contribute to the overall optical properties of the tegument, affecting the real and imaginary parts of the refractive index to different extents. While pigments produce colour by absorbing specific wavelengths, when they are embedded within a matrix, they alter the local structure and its physical properties, including the refractive index. Conversely, the properties of the pigments depend on the chemical environment and hence on the matrix. In this sense, it can be said that the effects of structure and pigments do not merely add up, but rather interact.

Given these considerations, we herein revisit the commonly held belief that the colouration of ladybirds’ elytra is due to pigments, particularly carotenoids and melanin. We focused on two species of ladybirds: *A. bipunctata* and *C. quatuordecimguttata*.

Our empirical results demonstrate that pigments, including carotenoids and/or melanin, are present in both species. These pigments may be embedded within the chitin of the entire elytron or reside in specific layers within the chitin matrix, as observed for the black spots of *A. bipunctata* and the brown zones of *C. quatuordecimguttata*, respectively. Indeed, the TEM analysis revealed the presence of layers with dark contrast in the endocuticle of these regions, which are absent in the red or creamy zones of the elytra. This darker contrast likely results from the presence of an electron-absorbing molecule, presumably a pigment, suggesting that differences in colours within an elytron are determined by pigments distribution. This supports our hypothesis that pigments modulate the structural colouration of the elytron. Notably, except for the presence of the dark contrast layer, the microstructure is consistent across all zones of the elytron (spots and ground colour) in each species, indicating that a single physical mechanism governs wavelength selection in each species.

The black spots of *A. bipunctata* are associated with the presence of eumelanin, as identified by Raman spectroscopy and producing the typical dark contrast in TEM cross sectional images. Eumelanin absorbs all wavelengths across the visible spectrum, irrespective of the structural selection of wavelength, making it the primary contributor to the black pigmentation. In contrast, we were unable to identify, by chemical analyses and Raman spectroscopy, the pigment producing the dark contrast in TEM images of *C. quatuordecimguttata* brown zones remains unidentified through both chemical analyses and Raman spectroscopy. A potential candidate is eumelanin, which may be present at a lower density compared to the black spots of *A. bipunctata*, thereby accounting for the reduced contrast. Alternatively, pheomelanin, a form of melanin that produces reddish-brown to yellow pigmentation, could be involved, as it is the case for certain insect species [43]. However, its characteristic Raman peaks centred at 500, 1490, and 2000 cm⁻¹ [42] are conspicuously absent from the spectra obtained from all regions of the elytra of both species. Another possible candidate is a pigment from the pteridine family, responsible for yellow, orange or red colouration, which has recently been shown to be synthesised by insects [44]. However, we didn’t observe any fluorescence under UV light, which is characteristic of pteridines.

With regard to carotenoids, TEM did not reveal any specific layers with contrast variations indicative of carotenoid presence. High-magnification analysis of the exocuticle multilayer in both species revealed nanoparticles with diameters between 25 nm and 40 nm, showing a darker contrast than the surrounding matrix, suggesting they may represent pigment aggregates. Raman spectroscopy revealed variations in carotene peak intensities between the two species, with a stronger signal detected in *A. bipunctata* compared to *C. quatuordecimguttata*, suggesting a higher concentration of carotenoids in the former species, corroborating chemical analyses. Additionally, Raman peaks from both the brown and creamy regions of *C. quatuordecimguttata* elytra exhibited similar intensities, implying that carotenoids do not account for the colour differences between the spots and the remaining elytron. Notably, no known pigment is capable of producing the shiny white/cream colour of the spots in *C. quatuordecimguttata*, suggesting that this colouration is not pigment-based.

Other pigments may be present in the elytra of both species, though Raman detection can be limited, especially for pigments in deeper layers. The technique is more sensitive to molecules with many non-polar bonds, like carotenoids, while pigments with fewer non-polar bonds are less detectable. Thus, some pigments may evade detection if their concentrations are below the Raman threshold or masked by carotenoids.

However, if pigments are responsible for modulating colour across different zones of the elytra in the two studied species, the fundamental question remains: what are the physical mechanisms governing the interaction between light and the elytra structure, in conjunction with pigments, that produce the final perceived colour? It is important to note that light interaction with the elytra structure may involve different physical mechanisms, which must be considered prior to assessing the origin of colouration in ladybirds.

The two primary physical mechanisms responsible for colouration in biological tissues are incoherent scattering (or diffusion) and coherent scattering (i.e., interference or diffraction). The operation of these mechanisms within the elytral structure and the specific structural elements involved are described below.

Structural non-iridescent colouration generally arises from light diffusion by particles [45] which weakly scatters red wavelengths, making red colouration difficult through diffusion alone. When scattering conditions favours red wavelengths, other visible wavelengths diffuse, resulting in a white/creamy appearance. The scattering efficiency of different visible wavelengths is discussed in greater detail in [46].

The smaller concavities observed on the surface of the elytra of the species studied here may incoherently scatter light, producing a white or cream colour as seen in the scales of *Pieris rapae* (L.) (Lepidoptera), where a creamy hue results from scattering by densely packed pterin granules (pterinosomes) [46]. In these butterflies, the size of the isolated particles is insufficient for optimal scattering, but their close packing leads to dependent scattering producing light diffusion across the visible spectrum and resulting in a white/creamy hue. In the elytra of both ladybird species, the concavities range from 100 nm to 400 nm, similar to the size of *P. rapae* granules, and could contribute to colour production by scattering light across all wavelengths. However, the density of concavities is higher in *C. quatuordecimguttata* than in *A. bipunctata*, making dependent scattering more likely in the former species. As a result, *C. quatuordecimguttata* is expected to exhibit more intense scattering effects compared to *A. bipunctata*, where this effect can be considered negligible.

In contrast to the previous diffusion mechanism, structural colours from coherent scattering depend on the optical path length and vary with the observer’s angle, typically resulting in iridescence. While iridescence is absent in the ladybird elytra, the pigments within the cuticle matrix may suppress it by absorbing specific reflected wavelengths. Therefore, the lack of iridescence does not exclude the potential for interferential colours. Cross sectional analysis of the elytra shows, for both species, a laminar structure characteristic of beetle elytra, with alternating dark and bright regions visible in TEM images. The endocuticle sublayers in both species are at least one micrometre thick, which is too thick to generate interferential colours within the visible range. However, the exocuticle in both species contains a chirped (i.e., with variable thickness) multilayer that we have shown to function as a reflector in the visible range, as it has already been observed in other beetles, notably in *Charidotella egregia* (Boheman) [20]. Indeed, our numerical simulations indicate for both the investigated ladybird’s species that the light interaction with these structures produces wavelength selection. The chirped multilayers in the exocuticle are interferential layers that are able to generate a structural colouration whose chromaticity depends on the average refractive index of the chitin matrix.

The elytral structure of ladybirds is complex and the light interactions that induce colour production can include surface scattering, interference from multilayers and absorption from embedded pigments. As a result, significant challenges exist in predicting perceived colour. One of such challenges is the need to estimate not only the real part of the refractive index but also the imaginary part, which is related to the level of pigment absorption. The primary challenge when predicting the colouration produced by an interferential multilayer, such as those in the ladybird exocuticle, lies in determining the average refractive index and the refractive indices of each sub-layer. While spectroscopic ellipsometry has been successfully employed to obtain complex refractive indices in other beetle species [47], conducting similar measurements on ladybird elytra is exceptionally difficult due to their morphology, as the curved surface distorts the elliptical shape of the beam, complicating the measurements. Previous studies on the colouration of beetle bodies or elytra have used average refractive indices for chitin to estimate the wavelength of maximum reflection. Indeed, although approximations are necessary, when the period is relatively constant, it is possible to roughly estimate the colour produced by multilayers, as suggested by Vigneron et al. [20]. For their calculations, these authors used the refractive index most commonly cited for chitin in recent literature, which is 1.56. However, this value may decrease depending on the water and air content within the chitin matrix. Furthermore, in the present study, which deals with chirped layers of increasing thickness, there is no simple method to determine the reflected colour, and numerical simulations were employed here to predict the full spectrum of reflected light. In contrast to previously referenced research, this study employed the FDTD method to achieve precise rather than approximate predictions of the optical response. The results of our simulations are presented as a function of the refractive index to address the problem of approximating its value. Moreover, by accounting for variations in the refractive index, we can simultaneously consider colour variations induced by density, air, and water in the matrix.

In this study, only the real part of the refractive index was considered, with the imaginary part assumed to be zero. To evaluate the imaginary component of the refractive index (i.e., the absorption contribution), future work will need to provide a more comprehensive understanding of the pigments present in the elytra and their concentrations. Our preliminary chemical investigations provide complementary insights into the pigmentary component of this complex system. The significant difference in beta-carotene content between *A. bipunctata* and *C. quatuordecimguttata*, alongside the clear metabolomic discrimination between these species, opens new perspectives for investigating the chemical basis of both matrix and pigments in Coccinellidae. This approach could reveal how pigment composition interacts with structural features to produce the final colour phenotype, potentially identifying new compounds involved in this interplay. Future studies combining metabolomics with structural analysis could provide a more comprehensive understanding of colour production in these insects.

On the basis of our findings and the discussion above, we propose the following scenario to explain the origin of colouration in ladybirds:

In A. bipunctata elytra, coherent scattering from the chirped multilayer structure is the primary mechanism for colour production. The chromaticity shifts from green to orange based on the refractive index, with orange hues occurring between 1.45 and 1.50, and green at a refractive index of 1.55, close to that of chitin. The perceived colour is further influenced by pigments, with carotenoids affecting the ground colour and melanin contributing to the black spots. When the refractive index induces green, which coincides with carotenoids’ absorption range, an orange hue is produced. If the refractive index favours an orange hue, carotenoids enhance this colour, intensifying the overall appearance. This suggests that for a fixed carotenoid content, the perceived colour intensity is influenced by the refractive index, which depends on the elytra’s density and composition, particularly its protein and water content. Eumelanin in the black spots absorbs all visible wavelengths, accounting for their black appearance.

In C. quatuordecimguttata, the chirped multilayer in the endocuticle produces hues in the light yellow to orange range, with a refractive index of 1.3 closely matching the creamy spots’ appearance. High-density surface cavities may also contribute to this hue by scattering light, suggesting both mechanisms could coexist. While carotenoids are found in low concentrations in the creamy and brown regions, they are not present in all individuals, implying they do not contribute significantly to the colour. The brown colouration may instead be linked to an unidentified pigment that absorbs visible light.

To summarise:

- Both structure and pigments contribute to ladybird elytra colouration.
- The chirped multilayer in both A. bipunctata and C. quatuordecimguttata reflects visible light to produce colour, as confirmed by FDTD simulations. Surface cavities in C. quatuordecimguttata may also scatter light.
- Carotenoids, melanins, and potentially unknown pigments modulate colour across different elytra regions.

Our results indicate that both the structure and pigments contribute to the cuticle’s hue, meaning that the study of ladybird colouration must consider the interaction between these factors. For instance, untapping the genetic basis of colouration will require examining genes affecting both the structural arrangement of the cuticle and pigment production [48]. More importantly, considering structure is crucial for understanding colour evolution. For example, the intensity or size of a colour patch is expected to be a function of carotenoid deposition and thus of the entailed costs, which supposedly ensures the honesty of colouration as a signal of an individual’s quality [2,3]. However, as structure influences colouration and, consequently, the signal sent to receivers (*e.g.*, potential predators), it may finally also affect signal honesty. This may explain why carotenoids and melanin fall short of explaining that the intra and interspecific relative toxicity of ladybirds with contrasting colours do not consistently align with their degree of warning colouration [49–51]. Recognising the role of cuticle structure in colouration may open avenues to better understand how toxicity and colourations are linked.

## Conclusion

The structure of the ladybird elytra and not only pigments contributes to the overall colour. We identified two distinct physical mechanisms for colour production, namely coherent scattering from chirped multilayers and incoherent scattering from small concavities on the elytron surface. However, other structures and physical mechanisms may exist in different ladybird species and should be analysed in order to gain a deeper understanding of colour production. In addition, modelling all the interactions between the tegument and light require a more precise knowledge of its optical properties, specifically the real and imaginary components of the refraction index, resulting in a more accurate assessment of the various structural and pigmentary contributions to the reflectance spectrum. A consequence of this work is that ecological studies related to colour should include both structural aspects and the genetic factors related to structure.

## Supporting information

S1 File Colour chart reproduction. (PDF)

S2 File Experimental section chemistry. (PDF)
